# Supplementary material for: A digital twin for DNA data storage based on comprehensive quantification of errors and biases
Source: Nat Commun. 2023 Sep 27;14:6026. doi: 10.1038/s41467-023-41729-1 (PMC10533828; doi:10.1038/s41467-023-41729-1)
Supplement: Supplementary file 2 — Reporting Summary [file 41467_2023_41729_MOESM2_ESM.pdf]

nature portfolio

Corresponding author(s): Robert N. Grass
Last updated by author(s): Sep 14, 2023

Reporting Summary

Nature Portfolio wishes to improve the reproducibility of the work that we publish. This form provides structure for consistency and transparency in reporting. For further information on Nature Portfolio policies, see our Editorial Policies and the Editorial Policy Checklist.

Statistics

For all statistical analyses, confirm that the following items are present in the figure legend, table legend, main text, or Methods section.

n/a	Confirmed	
☐	☒	The exact sample size (*n*) for each experimental group/condition, given as a discrete number and unit of measurement
☒	☐	A statement on whether measurements were taken from distinct samples or whether the same sample was measured repeatedly
☐	☒	The statistical test(s) used AND whether they are one- or two-sided<br>*Only common tests should be described solely by name; describe more complex techniques in the Methods section.*
☒	☐	A description of all covariates tested
☐	☒	A description of any assumptions or corrections, such as tests of normality and adjustment for multiple comparisons
☐	☒	A full description of the statistical parameters including central tendency (e.g. means) or other basic estimates (e.g. regression coefficient) AND variation (e.g. standard deviation) or associated estimates of uncertainty (e.g. confidence intervals)
☐	☒	For null hypothesis testing, the test statistic (e.g. *F*, *t*, *r*) with confidence intervals, effect sizes, degrees of freedom and *P* value noted<br>*Give P values as exact values whenever suitable.*
☒	☐	For Bayesian analysis, information on the choice of priors and Markov chain Monte Carlo settings
☒	☐	For hierarchical and complex designs, identification of the appropriate level for tests and full reporting of outcomes
☒	☐	Estimates of effect sizes (e.g. Cohen's *d*, Pearson's *r*), indicating how they were calculated

Our web collection on statistics for biologists contains articles on many of the points above.

Software and code

Policy information about availability of computer code

Data collection	Custom software based on Python (v3.10) and its libraries RapidFuzz (v2.6) and edlib (v1.2) was used for read alignment, error analysis, and generation of simulated sequencing data. The custom software is available at https://github.com/fml-ethz/dt4dds. BBMap (v38.99) was used for collecting data on sequence coverage from sequencing data.
Data analysis	Custom scipts based on Python (v3.10) were used for analyzing and plotting data. The scripts are available at https://github.com/fml-ethz/dt4dds_notebooks.

For manuscripts utilizing custom algorithms or software that are central to the research but not yet described in published literature, software must be made available to editors and reviewers. We strongly encourage code deposition in a community repository (e.g. GitHub). See the Nature Portfolio guidelines for submitting code & software for further information.

Data

Policy information about availability of data

All manuscripts must include a data availability statement. This statement should provide the following information, where applicable:

- Accession codes, unique identifiers, or web links for publicly available datasets
- A description of any restrictions on data availability
- For clinical datasets or third party data, please ensure that the statement adheres to our policy

The experimental and simulated sequencing data generated in this study have been deposited in the European Nucleotide Archive under accession code PRJEB65931. Sequencing data from the literature used for analysis is available from the studies by Koch et. al. (PRJEB35217), Erlich et. al. (PRJEB19305 and

PRJEB19307), and Chen et. al. (github.com/uwmisl/storage-biasing-ncomms20)

Research involving human participants, their data, or biological material

Policy information about studies with human participants or human data. See also policy information about sex, gender (identity/presentation), and sexual orientation and race, ethnicity and racism.

Reporting on sex and gender	Study did not consider human participants, their data, or their biological material.
Reporting on race, ethnicity, or other socially relevant groupings	Study did not consider human participants, their data, or their biological material.
Population characteristics	Study did not consider human participants, their data, or their biological material.
Recruitment	Study did not consider human participants, their data, or their biological material.
Ethics oversight	Study did not consider human participants, their data, or their biological material.

Note that full information on the approval of the study protocol must also be provided in the manuscript.

Field-specific reporting

Please select the one below that is the best fit for your research. If you are not sure, read the appropriate sections before making your selection.

[x] Life sciences [ ] Behavioural & social sciences [ ] Ecological, evolutionary & environmental sciences

For a reference copy of the document with all sections, see nature.com/documents/nr-reporting-summary-flat.pdf

Life sciences study design

All studies must disclose on these points even when the disclosure is negative.

Sample size	The experiments considered four different oligonucleotide pools, of which two each were from different commercial providers and two each were constrained to exactly 50% GC content. In all cases, the number of sequences in each oligonucleotide pool were chosen based on the maximum number offered by the synthesis provider at the respective synthesis scale. These sample choices cover the main synthesis providers and sequence design commonly used in literature. A sequencing coverage of around 90 reads per sequence was targeted in each sequencing experiment, in order to achieve reliable error statistics. At these high sequencing coverages, no stochastic effects are anticipated.
Data exclusions	No sequencing dataset was excluded from analysis. For the analysis of errors in the PhiX-based sequencing datasets, only the bases up to the length of the co-sequenced sample oligonucleotides were considered, as low diversity during base calling afterwards limits accuracy. For the same reason, the base positions corresponding to the index region of the co-sequenced sample oligonucleotides were also excluded.
Replication	Replication was achieved by performing the analysis independently with four different oligonucleotide pools by two different commercial suppliers, across multiple sequencing runs.
Randomization	No randomization was performed in this study, due to the type of experiments and analysis performed. Specifically, samples were not allocated into experimental groups such that no randomization was required.
Blinding	No blinding was performed in this study, due to the type of experiments and analysis performed. Specifically, no allocation into experimental groups was performed, such that blinding was not required.

Reporting for specific materials, systems and methods

We require information from authors about some types of materials, experimental systems and methods used in many studies. Here, indicate whether each material, system or method listed is relevant to your study. If you are not sure if a list item applies to your research, read the appropriate section before selecting a response.

Materials & experimental systems

n/a	Involved in the study
[x]	[ ] Antibodies
[x]	[ ] Eukaryotic cell lines
[x]	[ ] Palaeontology and archaeology
[x]	[ ] Animals and other organisms
[x]	[ ] Clinical data
[x]	[ ] Dual use research of concern
[x]	[ ] Plants

Methods

n/a	Involved in the study
[x]	[ ] ChIP-seq
[x]	[ ] Flow cytometry
[x]	[ ] MRI-based neuroimaging
